# Antihypertensive drug use and breast cancer risk: a meta-analysis of observational studies

**DOI:** 10.18632/oncotarget.19117

**Published:** 2017-07-10

**Authors:** Haibo Ni, Qin Rui, Xiaojue Zhu, Zhenquan Yu, Rong Gao, Huixiang Liu

**Affiliations:** ^1^ Department of Neurosurgery, The First People’s Hospital of Zhangjiagang City, Suzhou, Jiangsu, China; ^2^ Department of Laboratory, The First People’s Hospital of Zhangjiagang City, Suzhou, Jiangsu, China; ^3^ Department of Neurosurgery, The First Affiliated Hospital of Soochow University, Suzhou, Jiangsu, China

**Keywords:** antihypertensive drug, breast cancer, meta-analysis, cancer prevention, epidemiology

## Abstract

We conducted a meta-analysis of observational studies to examine the hypothesized association between breast cancer and antihypertensive drug (AHT) use. Fixed- or random- effect models were used to calculate pooled risk ratios (RRs) and 95% confidence intervals (CIs) for all AHTs and individual classes (i.e., angiotensin-converting enzyme inhibitors, [ACEi]; angiotensin-receptor blockers, [ARBs]; calcium channel blockers, [CCBs]; beta-blockers, [BBs], and diuretics). Twenty-one studies with 3,116,266 participants were included. Overall, AHT use was not significantly associated with breast cancer risk (RR = 1.02, 95% CI: 0.98-1.06), and no consistent association was found for specific AHT classes with pooled RRs of 1.02 (95% CI: 0.96-1.09) for BBs, 1.07 (95% CI: 0.99-1.16) for CCBs, 0.99 (95% CI: 0.93-1.05) for ACEi/ARBs, and 1.05 (95% CI: 0.99-1.12) for diuretics. When stratified by duration of use, there was a significantly reduced breast cancer risk for ACEi/ARB use ≥10 years (RR = 0.80, 95% CI: 0.67-0.95). Although there was no significant association between AHT use and breast cancer risk, there was a possible beneficial effect was found for long-term ACEi/ARB. Large, randomized controlled trials with long-term follow-up are needed to further test the effect of these medications on breast cancer risk.

## INTRODUCTION

Hypertension is a highly prevalent condition worldwide, affecting more than one billion individuals and causing 9.4 million deaths annually [1]. Antihypertensive drugs (AHTs) are commonly prescribed to help prevent detrimental outcomes of hypertension including stroke, coronary artery disease, and heart failure. It is estimated that AHT consumption has nearly doubled in OECD countries from 2000 to 2011. In the United States alone, the number of filled prescriptions reached 678.2 million in 2010 [2]. Despite their increasing use by patients with cardiovascular-related conditions, the noncardiovascular effects of AHTs remain unclear. Indeed, the carcinogenic potential of AHT has long been under scrutiny. During the past two decades, nearly all AHT classes have been reported to increase the risk of total cancer [3], as well as renal cancer [4], glioma [5], and epithelial ovarian cancer [6].

Breast cancer is the most common form of cancer and the second leading cause of cancer-related death among women worldwide [7]. There has been growing interest in the relationship between AHT use and breast cancer risk since the 1990s when Heinonen et al reported the results of a case-control study implicating rauwolfia derivatives in increasing breast cancer risk among women older than 50 [8]. Following this discovery, numerous observational studies examined the association between major AHT classes and breast cancer risk, but the results have been conflicting and inconsistent. Some groups [9–12] found that use of beta blockers (BBs), calcium channel blockers (CCBs), or diuretics was positively associated with breast cancer risk, but most [2, 13–24] observed no relationships. In addition, evidence for angiotensin-converting enzyme inhibitors or angiotensin receptor blockers (ACEi/ARBs) is also inconsistent, with some studies [2, 9, 12, 14, 16, 18, 20, 22, 25, 26] suggesting that their use is not associated with breast cancer risk, and others [23, 27] reporting increased or decreased risk.

Thus, given the widespread use of AHTs and the continued uncertainty regarding their effects on breast cancer incidence, we carried out a comprehensive meta-analysis to determine if there is an association of AHT use, including overall and different classes, with breast cancer risk based on all available observational studies.

## RESULTS

### Literature search

**Figure 1:**
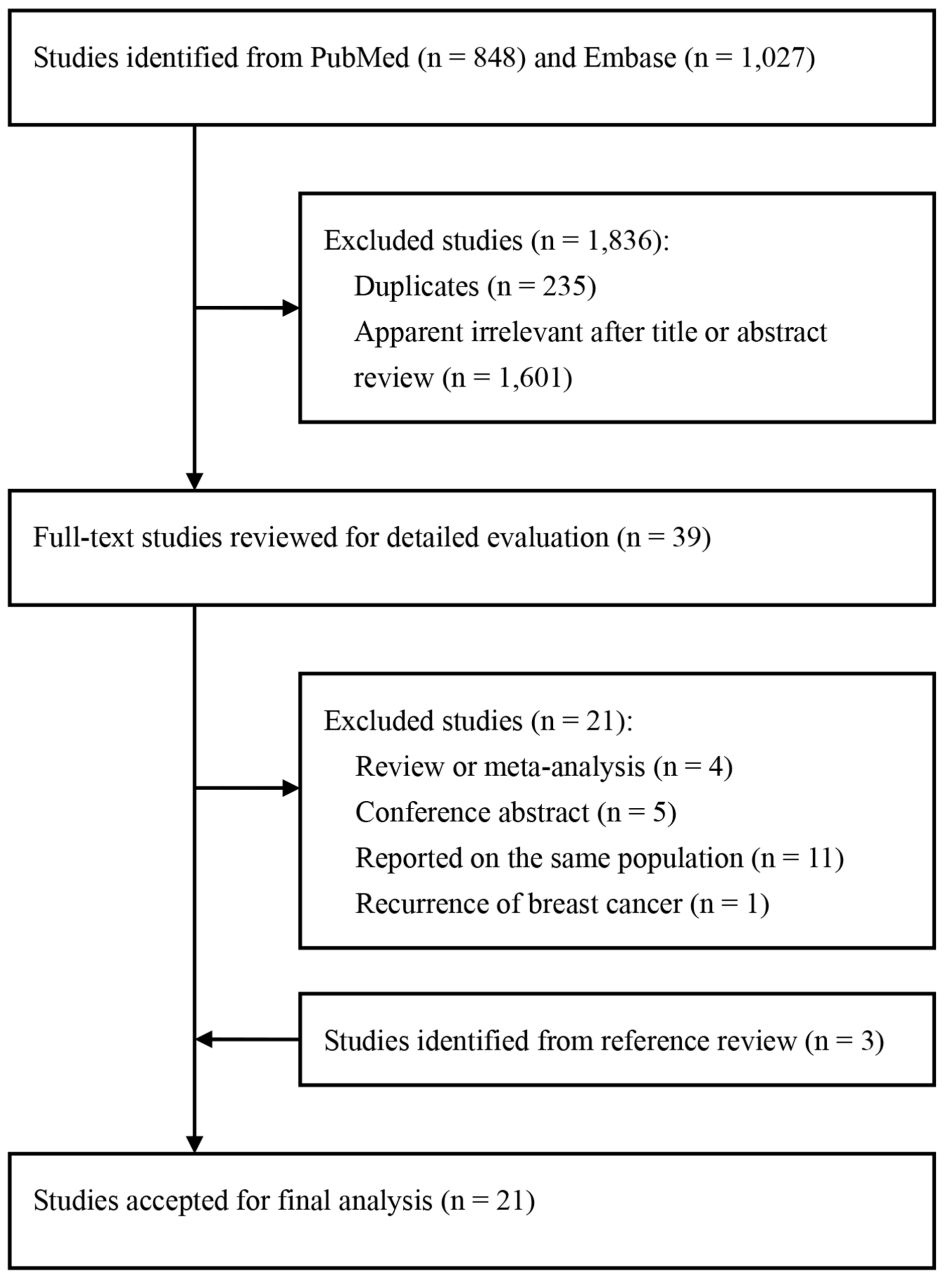
Flow chart of study selection

A total of 1,875 potentially eligible studies were identified during the initial search. After removing the duplicates and reviewing the titles or abstracts, 1,836 studies were deemed ineligible. Among the 39 articles for full-text review, 21 were further excluded for the following reasons: review or meta-analysis [32–35]; conference abstracts [36–40]; duplicate reports from the same study population [41–50]; or outcome was breast cancer recurrence [51]. Three additional articles [13, 14, 18] were included from the reference review. Finally, a total of 21 studies [2, 9–13, 15–27, 52, 53] published from 1996 to 2016 were included. The study selection process is depicted in Figure 1.

### Study characteristics

A list of details abstracted from the 21 included studies is provided in Table 1. All studies were published in English. Nine were prospective cohort studies, and 12 were case-control studies. Eleven studies were conducted in the United States, eight in Europe, one in Canada, and one in Taiwan. The sample sizes of the included studies ranged from 654 to 2,300,000, with a total of 3,167,020 participants, and the number of breast cancer cases varied from 31 to 58,000, with a total of 102,054. Of those studies, 11 provided results for BBs, 13 for CCBs, 13 for ACEi/ARBs, and 11 for diuretics. Drug use assessments were not consistent between studies; most used questionnaires and prescription database reviews. Case ascertainment was based on cancer registries or medical records in all studies. The adjusted covariates in individual studies differed, and most risk estimates were adjusted for age, body mass index, alcohol intake, and hormone replacement therapy use. Quality scores according to the Newcastle-Ottawa Quality Assessment Scale varied from 5 to 9 points, with a median of 7.14, indicating high quality of the studies included in the meta-analysis.

**Table 1:** Characteristics of observational studies of antihypertensive drug and breast cancer included in this meta-analysis

Author, year	Location	Study period/ follow-up (yrs)	Age (yrs)	No. of cases/ participants	Exposure variables	Exposure assessment	Case ascertainment	Adjustment for covariates	Quality score
Prospective studies									
Pahor et al [13], 1996	USA	1988-1992/3.7	≥71	31/3,256	CCBs	Self-administered questionnaire	Cancer registry	Age, race, hospitalizations, smoking, alcohol intake, oestrogen use, heart disease	7
Fryzek et al [16], 2006	Denmark	1990-2002/5.7	50–67	264/49,950	AHT, CCBs, BBs, ACEi/ARBs, and Diuretics	Prescription database	Cancer registry	Age, calendar year, age at first birth, parity, HRT, NSAID use	8
Van Der Knaap et al [25], 2008	Netherlands	1989-2004/9.6	≥55	142/4,710	ACEi/ARBs	Standard questionnaire	Cancer registry	Age, BMI, calendar year, physical activity, age at menarche and menopause, number of children, HRT, NSAID use, hypertension, diabetes, heart disease	8
Largent, et al [20], 2010	USA	1995-2006/10	52.8	5,865/188,291	AHT, CCBs, ACEi, and Diuretics	Self-administered questionnaire	Cancer registry	Age, BMI, race, physical activity, smoking, diabetes, drinking, age at first birth, menopausal status, number of children, breastfeeding, HRT, family history of breast cancer, hysterectomy	7
Biggar et al [10], 2013	Denmark	1995-2010	62.6	58,000/2,300,000	Spironolactone	Prescription database	Cancer registry	Age, calendar year	6
Saltzman et al [22], 2013	USA	1989-1993	≥65	188/3,201	AHT, CCBs, BBs, ACEi, and Diuretics	Self-administered questionnaire	Cancer registry	Age, income, waist-hip ratio, alcohol intake, age at menopause	7
Devore et al [23], 2015	USA	1988-2012	25-55	10,012/210,641	AHT, CCBs, BBs, ACEi, and Diuretics	Questionnaire	Medical records	Age, BMI, physical activity, height, shift work history, smoking, alcohol intake, age at menarche, menopause and first birth, parity, menopausal status, oral contraceptive use, HRT, family history of breast cancer, history of benign breast disease	8
Azoulay et al [24], 2016	UK	1995-2010/5.7	≥18	4,520/273,152	CCBs	Prescription database	Cancer registry	Age, BMI, calendar year, smoking, alcohol intake, oral contraceptive use, HRT use, NSAID use, aspirin, statins, hysterectomy, previous cancer	8
Wilson et al [53], 2016	USA	2003-2009/5.3	35-74	1,965/50,754	AHT, CCBs, BBs, ACEi/ARBs, and Diuretics	Standard questionnaire	Medical records	Age, BMI, race, physical activity, smoking, age at menarche, parity, menopausal status, HRT and statins use	9
**Retrospective studies**									
Rosenberg et al [52], 1998	USA	1976-1996	40-69	2,893/6,641	CCBs, BBs, and ACEi	Standard questionnaire	Medical records	Age, BMI, calendar year, smoking, alcohol intake, age at menarche, age at first birth, parity, age at menopause, oral contraceptive use, HRT use, family history of breast cancer, history of benign breast disease	8
Li et al [12], 2003	USA	1997-1999	65-79	975/1,982	AHT, CCBs, BBs, ACEi, and Diuretics	Standard questionnaire	Cancer registry	Age	6
Gonzalez-Perez et al [15], 2004	UK	1995-2001	30-79	3,780/23,780	AHT, and BBs	Prescription database	Medical records	Age, BMI, calendar year, smoking, alcohol intake, HRT use, use of other AHT, hypertension, prior breast lump	8
Largent et al [11], 2006	USA	1994-1995	50–75	523/654	Diuretics	Self-administered questionnaire	Cancer registry	Age, BMI, education, smoking, alcohol intake, age at first birth, menopausal status, diabetes, family history of breast cancer	6
Davis et al [17], 2007	USA	1992-1995	20–74	600/1,247	CCBs, and BBs	Telephone interview	Cancer registry	Smoking, alcohol intake, age at first birth, parity, oral contraceptive use, HRT, family history of breast cancer, hysterectomy, ever upper gastrointestinal series	5
Assimes et al [18], 2008	Canada	1978-1988	71.8	1,623/17,853	CCBs, BBs, and ACEi/ARBs	Prescription database	Cancer registry	Age, hypertension, diabetes, heart and chronic lung disease, cerebrovascular arterial disease, migraine, hyperthyroid, scleroderma, use of other AHT,	6
Coogan et al [19], 2009	USA	1976-2007	18-79	5,989/11,493	Diuretics	Standard questionnaire	Medical records	BMI, race, education, alcohol intake, parity, menopausal status, oestrogen and oral contraceptive use	8
Azoulay et al [26], 2012	UK	1995-2010/6.4	63.4	11,312/124,331	ACEi/ARBs	Prescription database	Cancer registry	BMI, smoking, alcohol intake, diabetes, oral contraceptive, HRT, hysterectomy, previous cancer, use of NSAID, aspirin, and statins	7
Mackenzie et al [21], 2012	UK	1987-2010/4.1	≥55	28,032/83,993	Spironolactone	Prescription database	Medical records	Age, BMI, calendar year, Townsend score, alcohol intake, oral contraceptive use, HRT, aspirin, finasteride, hypertension, diabetes, family history of breast cancer, history of benign breast disease, heart disease	6
Hallas et al [27], 2012	Denmark	2000-2005	69.4	19,947/332,623	ACEi/ARBs	Prescription database	Cancer registry	Oral contraceptive use, HRT use, NSAID use, aspirin, statins, finasteride, hypertension, diabetes, heart disease, inflammatory bowel disease, chronic lung and kidney disease	7
Li et al [2], 2013	USA	2000-2008	55–74	1,960/2,851	AHT, CCBs, BBs, ACEi/ARBs, and Diuretics	Standard questionnaire	Cancer registry	Age, calendar year, race, alcohol intake	7
Chang et al [9], 2016	Taiwan	2001-2011/9.9	≥55	9,397/46,985	DiCCBs, BBs, and ACEi/ARBs	Prescription database	Cancer registry	Socioeconomic status, Charlson’s index, number of hospitalizations and outpatient visits, hospital admission length, HRT use, aspirin, statins, fibrates, diuretics, human insulin, diabetes, heart disease, chronic kidney, liver, and lung disease, depression, cerebrovascular arterial disease, number of lipid measurements and mammography	8

AHT, antihypertensive drug; ACEi, angiotensin-converting enzyme inhibitors; ARBs, angiotensin receptor blockers; BBs, beta blockers; BMI, body mass index; CCBs, calcium channel blockers; DiCCBs, dihydropyridine calcium channel blockers; HRT, hormone replacement therapy; NSAID, non-steroidal anti-inflammatory drugs; UK, United Kingdom.

### Association between overall AHT use and breast cancer risk

**Figure 2:**
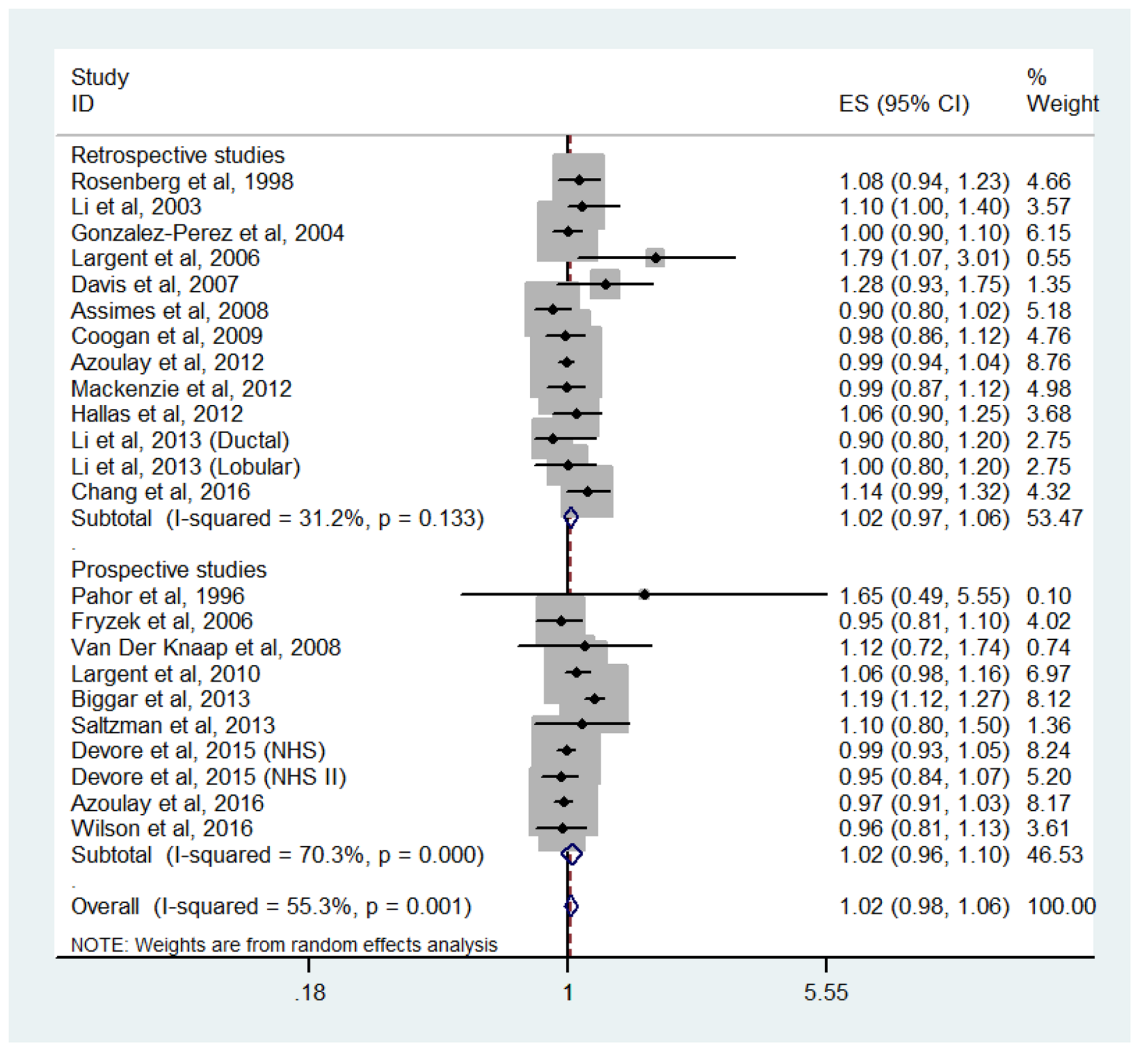
Forest plot of overall antihypertensive use and breast cancer risk CI, confidence interval; RR, relative risk.

Twenty-one epidemiologic studies (twelve retrospective and nine prospective) presented results on use versus nonuse of AHTs and breast cancer risk. The pooled RR was 1.02 (95% CI: 0.98-1.06), with moderate heterogeneity among studies (*P*_heterogeneity_ = 0.001, *I*^2^ = 55.3%; Figure 2). When stratified by study design, no significant association was found among retrospective studies (RR = 1.02, 95% CI: 0.97-1.06, *P*_heterogeneity_ = 0.133, *I*^2^ = 31.2%) or prospective studies (RR = 1.02, 95% CI: 0.96-1.10, *P*_heterogeneity_ = 0.000, *I*^2^ = 70.3%).

### Association between BB use and breast cancer risk

**Figure 3:**
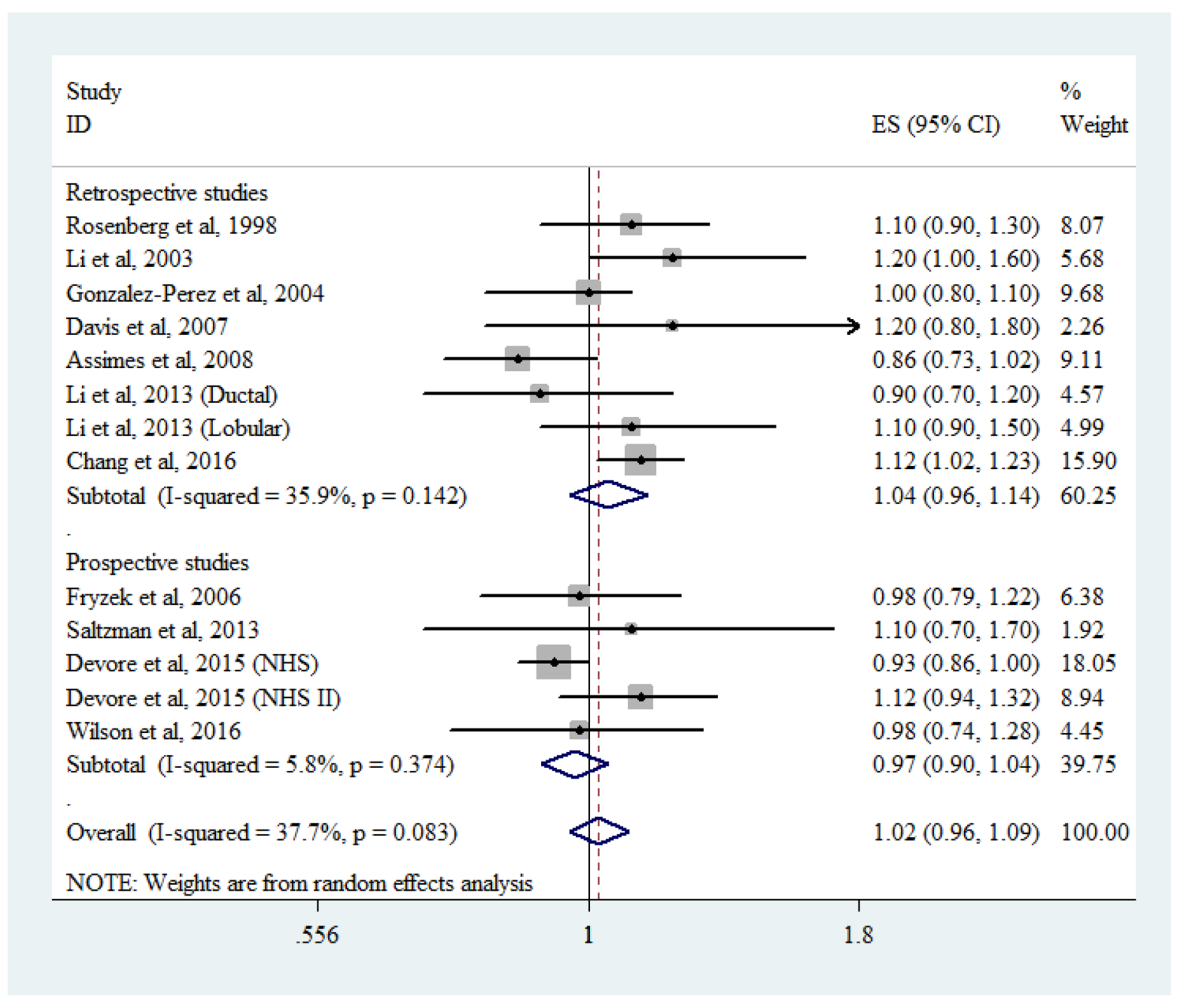
Forest plot of beta-blocker use and breast cancer risk CI, confidence interval; RR, relative risk.

An association between breast cancer risk and BB use was reported in 11 studies [2, 9–12, 15–18, 22, 23, 52, 53], including 7 retrospective studies and 4 prospective studies. The pooled RR was 1.04 (95% CI: 0.96-1.14, *P*_heterogeneity_ = 0.142, *I*^2^ = 35.9%) for retrospective studies and 0.97 (95% CI: 0.90-1.04, *P*_heterogeneity_ = 0.374, *I*^2^ = 5.8%) for prospective studies. Combining the retrospective and prospective data, the pooled RR was 1.02 (95% CI: 0.96-1.09) with low heterogeneity among all the studies (*P*_heterogeneity_ = 0.083, *I*^2^ = 37.7%; Figure 3).

### Association between CCB use and breast cancer risk

**Figure 4:**
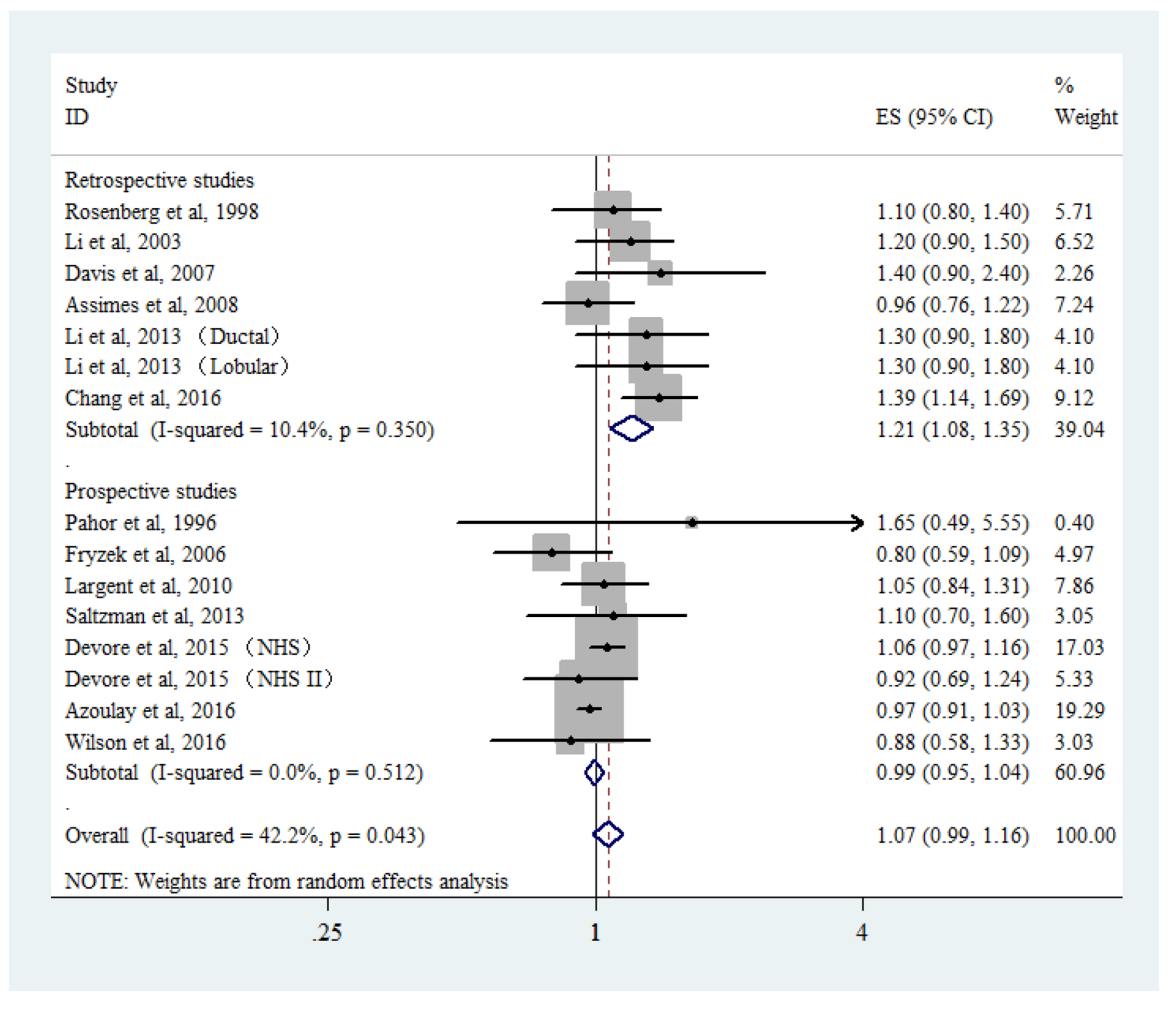
Forest plot of calcium channel blocker use and breast cancer risk CI, confidence interval; RR, relative risk.

Six retrospective studies and seven prospective studies were included in the analysis for breast cancer risk among CCB users. Low heterogeneity (*P*_heterogeneity_ = 0.043, *I*^2^ = 42.2%) was found among all the studies. Random-effects pooled analysis suggested that CCB use was not associated with breast cancer risk (RR = 1.07, 95% CI: 0.99-1.16; Figure 4). Subgroup analysis showed a positive association among retrospective studies (RR = 1.21, 95% CI: 1.08-1.35, *P*_heterogeneity_ = 0.350, *I*^2^ = 10.4%) but not among prospective studies (RR = 0.99, 95% CI: 0.95-1.04, *P*_heterogeneity_ = 0.512, *I*^2^ = 0.0%).

### Association between ACEi/ARB use and breast cancer risk

**Figure 5:**
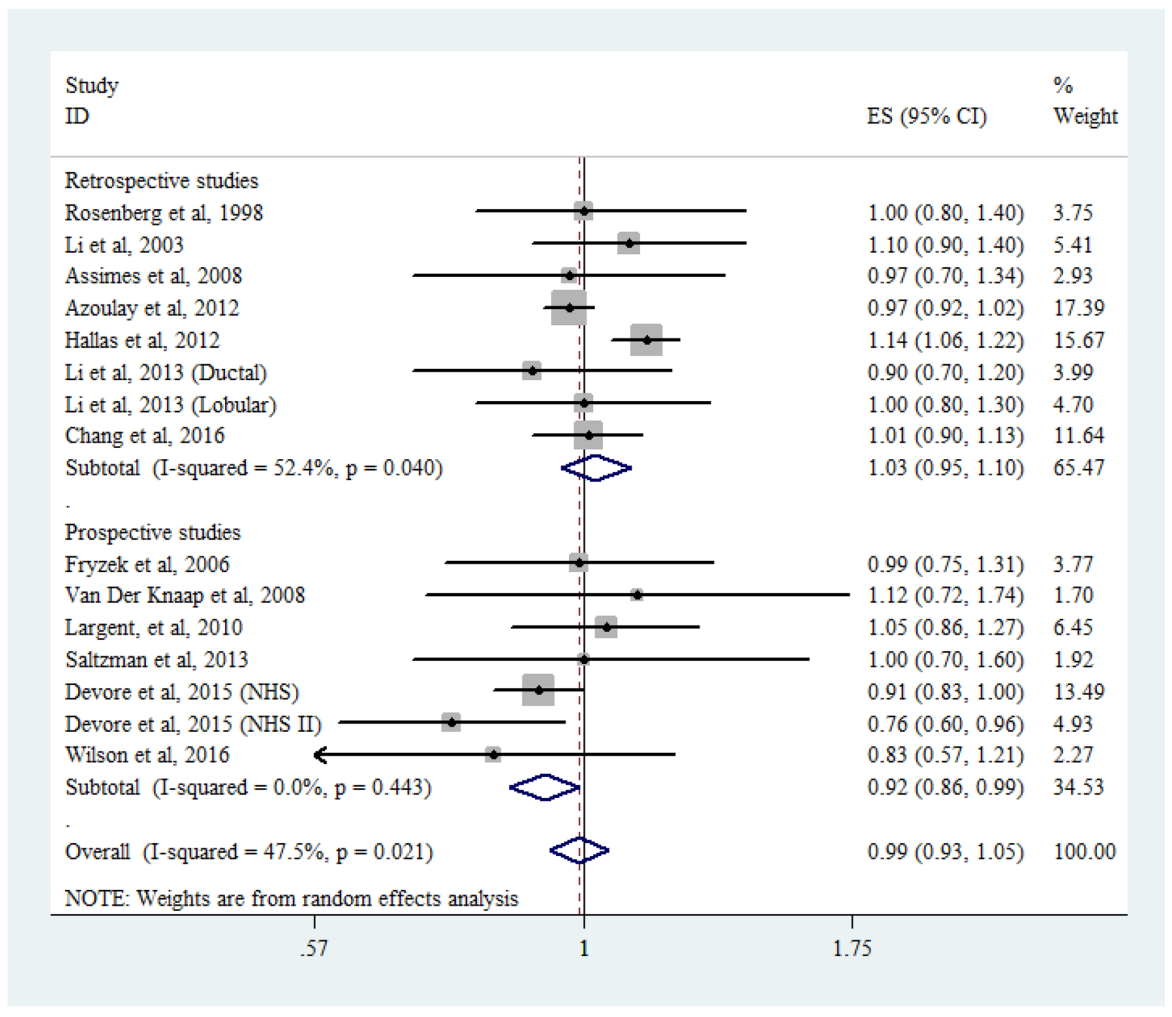
Forest plot of angiotensin-converting enzyme inhibitor/angiotensin-receptor blocker use and breast cancer risk CI, confidence interval; RR, relative risk.

Thirteen studies (seven retrospective and six prospective) examined the role of ACEi/ARB use on breast cancer risk. The results are shown in Figure 5. The pooled RRs comparing ACEi/ARB use and nonuse were 0.99 (95% CI: 0.93-1.05, *P*_heterogeneity_ = 0.021, *I*^2^ = 47.5%) for overall studies, 1.03 (95% CI: 0.95-1.10, *P*_heterogeneity_ = 0.040, *I*^2^ = 52.4%) for retrospective studies, and 0.92 (95% CI: 0.86-1.00, *P*_heterogeneity_ = 0.356, *I*^2^ = 9.3%) for prospective studies.

### Association between diuretic use and breast cancer risk

**Figure 6:**
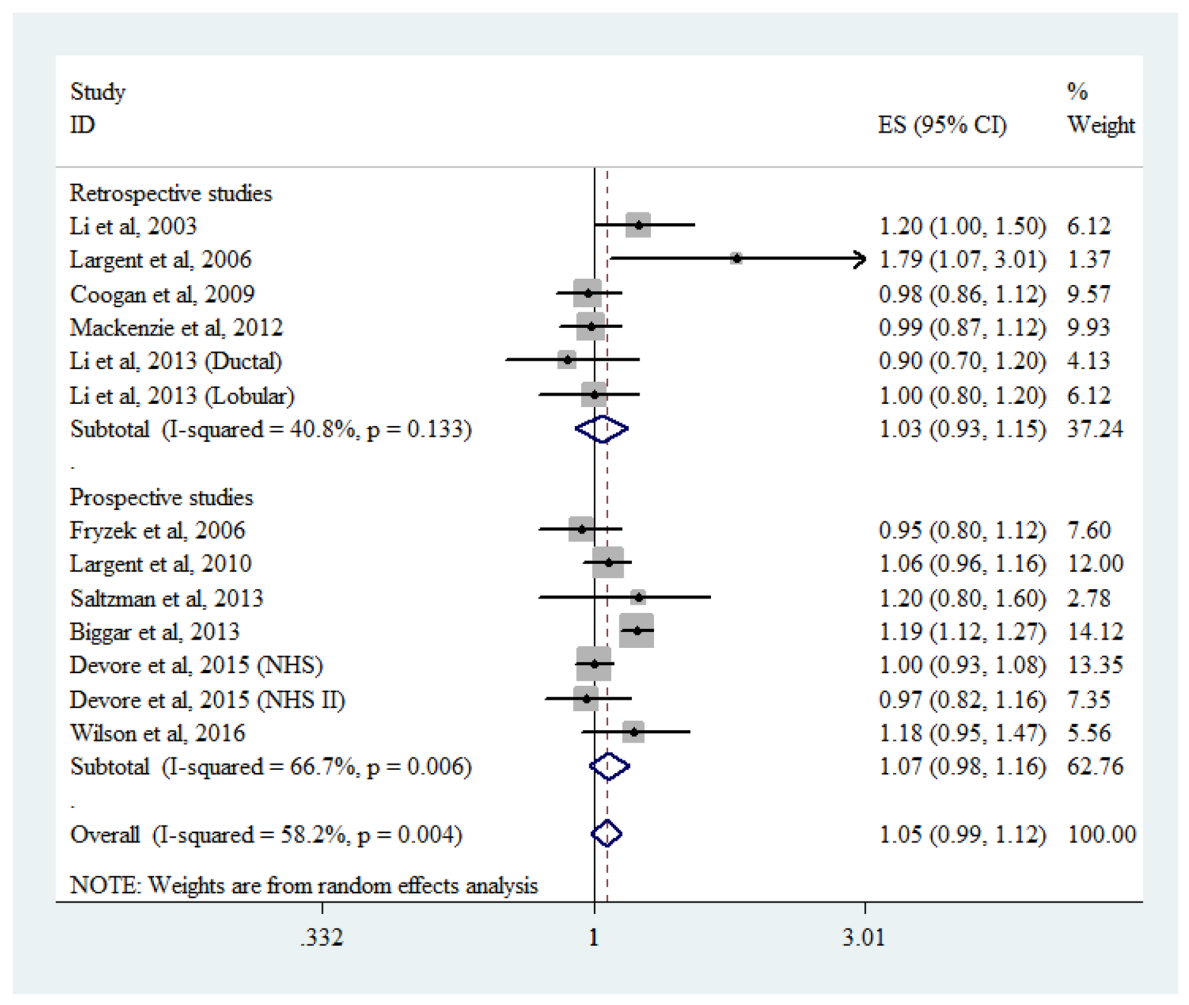
Forest plot of diuretic use and breast cancer risk CI, confidence interval; RR, relative risk.

Eleven studies provided information on diuretics use (Figure 6). Compared with nonuse, the pooled RR for diuretics was 1.05 (95% CI: 0.99-1.12). There was moderate heterogeneity across studies (*P*_heterogeneity_ = 0.004, *I*^2^ =58.2%). No significant link was found in retrospective studies (RR = 1.03, 95% CI: 0.93-1.15, *P*_heterogeneity_ = 0.133, *I*^2^ = 40.8%) or prospective studies (RR = 1.07, 95% CI: 0.98-1.16, *P*_heterogeneity_ = 0.006, *I*^2^ = 66.7%).

### Subgroup analyses

When stratifying by geographic region, we did not found any association between AHT use and breast cancer risk. There was also no association observed in either study quality score stratum. Stratification by time period of drug use (current, recent, or past) showed that exposure to any class of AHT did not alter breast cancer risk. However, in examining duration effects of medication use, a reduced risk of breast cancer was found for ACEi/ARB use for 10 years or longer (RR = 0.80, 95% CI: 0.67-0.95) but not in those observed for 5-10 years or fewer than 5 years. No statistically significant associations were seen for the other drug categories (Tables 2 and 3).

**Table 2:** Subgroup and sensitivity analyses of associations between use of overall AHT, BBs and CCBs and breast cancer risk

	AHT				BBs				CCBs			
Group	n	RR (95% CI)	*P*_heterogeneity_	*I*^2^ (%)	n	RR (95% CI)	*P*_heterogeneity_	*I*^2^ (%)	n	RR (95% CI)	*P*_heterogeneity_	*I*^2^ (%)
Total	21	1.02(0.98-1.06)	0.001	55.3	11	1.02(0.96-1.09)	0.083	37.7	13	1.07(0.99-1.16)	0.043	42.2
Design												
Retrospective study	12	1.02(0.97-1.06)	0.133	23.5	7	1.04(0.96-1.14)	0.142	35.9	6	1.21(1.08-1.35)	0.350	10.4
Prospective study	9	1.02(0.96-1.10)	0	70.3	4	0.97(0.90-1.04)	0.374	5.8	7	0.99(0.95-1.04)	0.512	0
Geographic area												
America	12	1.01(0.96-1.05)	0.176	25.9	8	1.01(0.93-1.09)	0.148	32.5	10	1.07(1.00-1.14)	0.745	0
Europe	8	1.02(0.96-1.10)	0	75.2	2	0.99(0.87-1.13)	0.883	0	2	0.94(0.81-1.08)	0.228	31.3
Study quality score												
High (NOS score >6)	15	1.00(0.97-1.02)	0.766	0	8	1.03(0.96-1.09)	0.167	30.3	10	1.06(0.97-1.16)	0.034	47.4
Low (NOS score ≤6)	6	1.10(0.96-1.25)	0	78.6	3	1.04(0.81-1.35)	0.046	67.5	3	1.11(0.91-1.35)	0.266	24.4
Time period of use												
Current use	6	1.06(0.95-1.19)	0	85.6	4	1.45(0.98-2.15)	0	95.9	4	1.72(0.96-3.09)	0	96.6
Recent use	4	1.07(0.98-1.17)	0.654	0	3	1.06(0.82-1.39)	0.257	26.3	4	1.16(0.98-1.36)	0.416	0
Former use	7	1.05(0.99-1.12)	0.209	26.4	5	1.00(0.95-1.06)	0.423	0	4	1.00(0.83-1.20)	0.037	60.9
Duration of use												
<5 years	10	0.99(0.95-1.03)	0.647	0	6	1.02(0.95-1.10)	0.595	0	6	1.00(0.90-1.10)	0.138	36.5
5-10 years	6	1.02(0.95-1.09)	0.683	0	4	0.94(0.84-1.06)	0.721	0	5	1.09(0.98-1.20)	0.985	0
≥10 years	7	1.01(0.92-1.12)	0.188	30.1	4	1.11(0.85-1.45)	0.016	67.2	5	1.07(0.71-1.60)	0.003	72.4
Exposure was defined as “ever use”	17	1.01(0.98-1.05)	0.175	24.2	7	1.06(0.96-1.16)	0.152	36.3	10	1.08(0.96-1.20)	0.027	52.2
Exposure was assessed by prescription database	9	1.02(0.95-1.09)	0	76.9	4	1.00(0.88-1.13)	0.049	61.9	4	1.02(0.84-1.24)	0.003	78.2

AHT, antihypertensive medications; BBs, beta blockers; CCBs, calcium channel blockers; RR, relative risk; CI, confidence interval.

**Table 3:** Subgroup and sensitivity analyses of associations between use of ACEi/ARBs and diuretics and breast cancer risk

	ACEi/ARBs				Diuretics			
Group	n	RR (95% CI)	*P*_heterogeneity_	*I*^2^ (%)	n	RR (95% CI)	*P*_heterogeneity_	*I*^2^ (%)
Total	13	0.99(0.93-1.05)	0.021	47.5	11	1.05(0.99-1.12)	0.004	58.2
Design								
Retrospective study	7	1.03(0.95-1.10)	0.040	52.4	5	1.03(0.93-1.15)	0.133	40.8
Prospective study	6	0.92(0.86-1.00)	0.356	9.3	6	1.07(0.98-1.16)	0.006	66.7
Geographic area								
North America	8	0.94(0.88-1.00)	0.547	0	8	1.04(0.98-1.10)	0.224	23.8
Europe	4	1.05(0.92-1.18)	0.004	77.6	3	1.05(0.90-1.23)	0.004	81.6
Study quality score								
High (NOS score >6)	11	0.98(0.92-1.05)	0.011	53.7	7	1.01(0.97-1.06)	0.693	0
Low (NOS score ≤6)	2	1.06(0.88-1.27)	0.530	0	4	1.15(0.99-1.33)	0.024	68.4
Time period of use								
Current use	4	1.05(0.85-1.30)	0	80.7	4	1.05(0.95-1.17)	0.003	72.0
Recent use	3	1.03(0.88-1.21)	0.380	0	1	1.20(0.80-1.90)	-	-
Former use	4	0.98(0.87-1.12)	0.097	49.0	4	1.03(0.89-1.19)	0.002	76.2
Duration of use								
<5 years	5	0.95(0.87-1.04)	0.713	0	6	1.03(0.97-1.10)	0.921	0
5-10 years	3	0.91(0.78-1.06)	0.596	0	4	0.99(0.89-1.09)	0.544	0
≥10 years	4	0.80(0.67-0.95)	0.610	0	5	1.09(0.99-1.19)	0.589	0
Exposure was defined as “ever use”	10	1.04(0.97-1.10)	0.120	36.0	7	1.05(0.97-1.13)	0.151	36.3
Exposure was assessed by prescription database	5	1.03(0.94-1.13)	0.009	70.2	3	1.05(0.90-1.23)	0.004	81.6

ACEi, angiotensin-converting enzyme inhibitors; ARBs, angiotensin receptor blockers; RR, relative risk; CI, confidence interval.

Further, we performed a subgroup analysis based on AHT subclasses. With respect to CCBs dihydropyridine and nondihydropyridines CCB use were assessed separately, but neither was associated with breast cancer risk. Similarly, analyses by specific ACEi/ARB type did not reveal any statistically significant association. However, in evaluating risks according to diuretic subclasses, a borderline elevated risk of breast cancer was observed among users of thiazides diuretics (RR = 1.12, 95% CI: 1.01-1.24) but not other diuretic subtypes (Table 4).

**Table 4:** Subgroup analyses of associations between particular types of antihypertensive drug use and breast cancer risk

Group	No. of studies	RR (95% CI)	*P*_heterogeneity_	*I*^2^ (%)
CCBs				
DiCCBs	5	1.07(0.90-1.27)	0.026	58.1
Non-DiCCBs	4	1.23(1.00-1.51)	0.115	43.5
ACEi/ARBs				
ACEi	10	0.98(0.91-1.06)	0.006	58.3
ARBs	5	1.02(0.96-1.08)	0.698	0
Diuretics				
Thiazides	5	1.12(1.01-1.24)	0.286	20.3
Loop	4	0.91(0.77-1.06)	0.481	0
Potassium sparing	6	1.17(1.00-1.36)	0.024	61.2

CCBs, calcium channel blockers; DiCCBs, dihydropyridine calcium channel blockers; Non-DiCCBs, Non-dihydropyridines; ACEi, angiotensin-converting enzyme inhibitors; ARBs, angiotensin receptor blockers; RR, relative risk; CI, confidence interval.

### Sensitivity analyses

To confirm the robustness of our results, we carried out several sensitivity analyses. First, we excluded four studies [2, 10, 15, 23] that defined exposure as current use (in contrast to ever use in most studies). Exclusion of these studies did not substantially alter the overall result. Second, we restricted our analyses to studies [9, 10, 15, 16, 18, 21, 24, 26, 27] that used prescription databases, which made them less susceptible than questionnaire-based studies to recall bias. Again, the risk estimates were firmly in line with the complete analysis (Tables 2, 3). Third, sensitivity analysis was performed for each drug category by sequential omission of individual studies using the random-effects model. The results revealed that no study appeared to influence the overall pooled risk estimates (data not shown). Notably, the study by Chang et al [9] may be the key contributor to the between-study heterogeneity for BBs and CCBs. After excluding the study, no evidence of heterogeneity was observed among the remaining studies for BBs (*P*_heterogeneity_ = 0.269, *I*^2^ = 17.8%) or CCBs (*P*_heterogeneity_ = 0.323, *I*^2^ = 11.8%).

### Publication bias

There was no evidence of publication bias with regard to use of overall AHTs or individual classes in relation to breast cancer risk according to Begg’s funnel plot and Egger’s regression test (*P* = 0.827 for AHTs, *P* = 0.396 for BBs, *P* = 0.127 for CCBs, *P* = 0.587 for ACEi/ARBs, and *P* = 0.734 for diuretics).

## DISCUSSION

Results from 21 observational studies including 3,167,020 participants and 102,054 cases show that there is no increase in breast cancer risk among users of AHTs overall or specific major classes as compared to nonusers. These findings remained consistent in most subgroup and sensitivity analyses, which considered study design, geographic area, time period of use, subtypes, drug exposure definition, and drug exposure assessment method. Yet, when stratified by duration of use, a significant reduced risk of breast cancer was particularly observed among females taking ACEi/ARBs for 10 years or longer.

In line with our findings, a network meta-analysis of randomized trials also showed no increased cancer risk with the use of CCBs, ACEi, ARBs, BB, or diuretics [54]. However, our study differs from that of Bangalore and colleagues [54] in that our main analyses specifically focused on the association between AHT use and breast cancer risk, which has a distinctive etiology and pathogenesis compared with other types of cancer. Moreover, the trial evidence of that meta-analysis [54] had a mean follow-up of only 3.5 years, suggesting that the exposure time to AHTs might have been insufficient to make any meaningful conclusions about cancer incidence in humans. By using observational studies in our meta-analysis, we were able to include studies with longer duration of drug use and conduct a subgroup analysis of studies with drug use for 10 years or longer.

CCB use has long been hypothesized to promote cell proliferation and tumor growth [13], yet epidemiological studies have reported mixed results in relation to breast cancer occurrence [2, 9, 12–14, 16–18, 20, 22–24]. Our study is generally consistent with two previous meta-analyses of observational data published in 2014 [55, 56], indicating no carcinogenic effect of CCB on breast cancer. In evaluating the effect of long-term CCB use, however, previous meta-analyses [55, 56] drew conflicting conclusions with both positive and null associations. This difference was likely due to the small number of included studies with data on duration ≥10 years (3 [55] and 2 [56], respectively) and insufficient statistical power in their analyses. Three large cohort studies of high quality (all NOS >7) have been published since the meta-analyses, and all showed no association with breast cancer incidence [23, 24, 53]. We added these updated studies to our analysis, which significantly increased the sample size and made our results more accurate. In the subgroup analysis, we found a positive association between CCB use and breast cancer risk in retrospective but not prospective studies. This difference is likely attributable to recall and selection bias inherent in retrospective design. Thus, the positive result should not be overemphasized. Taken together, our findings do not support an overall association of CCB use, including long-term use, with breast cancer risk.

Although our results provided no evidence of an overall association between ACEi/ARB use and breast cancer risk, a potentially intriguing finding is the decreased risk for longer duration of ACEi/ARB use (≥10 years). This finding is consistent with a prior Seattle-Puget Sound case-control study, which identified a borderline significant risk reduction for lobular breast cancers among women using ACEis for 10 years or longer (RR = 0.6, 95% CI: 0.4-1.0) [2]. Furthermore, in line with our finding, two nationwide prospective studies in Taiwan also demonstrated that the effect of ARBs on cancer prevention correlated with treatment duration [48, 57]. The potential mechanisms underlying this antineoplastic effect of ACEi/ARBs on breast cancer are manifold and not completely understood. Several *in vitro* studies have shown that ACEi/ARBs suppress the cell proliferative effects of angiotensin II in breast cancer by inhibiting the renin-angiotensin system and its downstream signaling proteins such as tissue factor, vascular endothelial growth factor (VEGF), and the transcription factors NF-κB and CREB [58–60]. ACEi/ARBs have also been implicated in inhibiting breast cancer adhesion and invasion through reducing expression of integrin subtypes α3 and β1 [61]. In addition, ARB use has been shown to prevent tumor growth and angiogenesis by blocking VEGF-A expression in mice models of breast cancer [62].

Preclinical studies have shown that antagonism of β-adrenergic receptor signaling by BBs may inhibit multiple cellular processes involved in breast cancer initiation and progression, including cell proliferation, angiogenesis, and tumor immune responses [63]. While a few studies have reported associations between BB use and breast cancer risk [9, 50], our findings are consistent with the majority of observational studies that found no effect of BBs. However, we were unable to explore the relationship between the use of particular types of BBs and breast cancer risk since most of the studies reported BB as a composite class of AHTs and did not separately report the effects of beta-1 selective and nonselective subtypes. Only one case-control study in Taiwan [50] addressed this point and showed an increased risk for treatment with beta-1 selective blockers but not nonselective blockers. Therefore, whether the association differs according to BB subtype warrants further study.

With respect to diuretics, we did not observe an increased risk of breast cancer associated with overall diuretic use. Moreover, no trend of increasing risk with increasing duration of use was observed. Of note though, our subgroup analyses did show that use of thiazide diuretics but not other diuretic subclasses was significantly associated with an increased risk of breast cancer. To interpret the difference by drug subtype is challenging. One possible explanation is that thiazide diuretic use may increase insulin resistance [64], which has long been suggested as a risk factor for breast cancer [65, 66]. Alternatively, the borderline significant association may have occurred by chance due to the limited number of studies and participants analyzed. Consequently, this observation needs to be interpreted cautiously, and it requires replication in studies with sufficient numbers of specific diuretic subtype users.

Even though most of the included studies in this meta-analysis were of high quality as evidenced by high Newcastle-Ottawa quality assessment scores, we acknowledge that there were some limitations, and thus, the results should be interpreted with caution. First, this was a meta-analysis of observational studies, which are inherently prone to several types of bias [67]. For example, most AHT users are hypertensive, leading to selection bias of an unhealthier exposed group. These subjects might also undergo more medical examinations and laboratory surveillance, resulting in detection bias. Additionally, since ascertainment of AHT use largely depended on questionnaires, there is potential for recall bias, and exposure misclassification may have occurred. Second, most of the included studies (except for that by Chang et al [9]) were conducted in Western populations. Therefore, the results might not be generalizable to other groups, especially Asian AHT users with a different baseline breast cancer risk. Third, significant heterogeneity was observed among studies of individual classes of AHT and breast cancer risk. This persisted despite stratifying the data into subgroups based on study design, region, drug class, time period, and duration of drug use. Fourth, confounders were not uniformly adjusted across the included studies. Therefore, we cannot exclude the possibility that potential confounders such as body mass index, diabetes, alcohol use, chronic liver disease, and kidney disease involved in AHT metabolism may have affected the associations. Finally, publication bias could be of concern in our meta-analysis, although no evidence of such a bias was found with Begg’s funnel plot or Egger’s test. However, the number of studies included was relatively small, which may limit their statistical power.

In conclusion, the results of our study suggest a possible beneficial effect of long-term ACEi/ARB use on breast cancer risk. Considering potential biases and confounders in this meta-analysis of observational studies, large clinical trials with long-term follow-up are needed to fully assess the effect of these medications on breast cancer risk.

## MATERIALS AND METHODS

### Literature search

A comprehensive, computerized literature search was independently performed by two investigators (Q.R. and H.B.N.) in PubMed and EMBASE databases from January 1966 through July 2016. The following text and/or medical subject heading terms were used: “antihypertensive drug” or “calcium channel blockers” or “beta blockers” or “angiotensin-converting enzyme inhibitors” or “angiotensin receptor blockers” or “diuretics” combined with “breast cancer” or “breast neoplasm.” In addition, the reference lists of reviews and retrieved articles were manually searched to identify additional relevant articles. No language restrictions were imposed. The present study was performed in accordance with the guidelines proposed by the Meta-analysis of Observational Studies in Epidemiology group [28].

### Study selection

Studies were eligible for this meta-analysis if they fulfilled the following inclusion criteria: (1) published as an original article; (2) used a case-control or cohort design; (3) the exposure of interest was AHT intake, including the following five classes: ACEi, ARB, CCB, BB, or diuretics; (4) outcome was primary breast cancer occurrence; and (5) reported relative risk (RR), odds ratio (OR), or hazard ratio (HR) with corresponding 95% confidence intervals (CIs) or sufficient data to calculate them. When multiple studies reported the same data, results from the publication including the largest number of participants were used. We did not consider conference abstracts for inclusion.

### Data extraction and quality assessment

From each included study, the following information were recorded: first author’s surname, publication year, study design, geographical location, study period, duration of follow-up evaluation in cohort studies, participant age, numbers of cases and participants, type of medication exposure, assessment method of exposure and breast cancer, and adjustments for confounders. We extracted the risk estimates that reflected the greatest degree of control for potential confounders from each eligible study.

The Newcastle-Ottawa scale was used to assess the quality of individual studies. In brief, a maximum of 9 points was assigned to each study: 4 for selection, 2 for comparability, and 3 for outcomes. A final score >6 was regarded as high quality. Data extraction and quality assessment were performed by two independent investigators (Q.R. and H.B.N.). Any disagreement was settled by discussion.

### Statistical analysis

We used RRs as common measures of the association between AHT use and breast cancer risk across studies. For one study [23] that stratified risk estimates by two subcohorts (NHS and NHS II) and another study [2] that reported stratified risk estimates by tumor subtype (ductal and lobular breast cancer), we treated each result as a separate report. The combined risk estimates were computed using either a fixed-effect model or, in the presence of heterogeneity, a random-effect model. Between-study heterogeneity was evaluated by Cochran’s *Q* and *I*^*2*^ statistics. For Cochran’s *Q* statistic, results were defined as heterogeneous for *P* values less than 0.10; *I*^*2*^ values of 25%, 50%, and 75% represented cut-off points for low, moderate, and high heterogeneity, respectively [29].

We estimated the associations between overall AHT use as well as specific classes (CCB, ACEi/ARB, BB, and diuretics) and breast cancer risk. For six studies [9, 17, 18, 26, 27, 52] that only reported stratified risk estimates by AHT subtype, we combined the estimates using a random-effects model and then included the pooled estimates in the overall AHT meta-analysis. Among included studies, the most common definition of drugs exposure was “ever use vs. never use,” although four studies [2, 10, 15, 23] only provided results for “current use vs. never use,” we included all these studies in the main meta-analysis and performed a sensitivity analysis that only included studies with exposure defined as “ever use vs. never use.” Prespecified subgroup analyses were performed according to study design (retrospective or prospective), geographic area (North America or Europe), study quality score (high or low), time period of drug use (current, recent, or former), duration of drug use (<5, 5-10, or ≥10 years), and subtype of individual classes to examine the impact of these factors on the associations. Current use was defined as AHT use that lasted until the index date or ended within 6 months prior to the index date, former use was defined as use that ended more than 6 months before the index date, and recent use was defined as use that ended within 2 years prior to the index date. Due to limited number of studies provided data on BB subtypes, the stratified analysis by subclasses focused on CCBs (dihydropyridine or nondihydropyridines), ACEi/ARBs, and diuretics (thiazides, loop, or potassium sparing). To test the robustness of associations, we performed a sensitivity analysis restricted to studies that used a prescription database to identify drug exposure. We also investigated the influence of a single study on the overall risk estimate by omitting each study in each turn.

Potential publication bias was examined using Begg’s funnel plots [30] and Egger’s regression tests [31]. All statistical analyses were performed using STATA 12.0 (Stata Corporation, College Station, TX, USA) statistical software. A *P* value less than 0.05 was considered statistically significant unless otherwise specified.
